# Patterns of Care and Outcomes of Adjuvant Radiotherapy for Meningiomas: A Surveillance, Epidemiology, and End Results and Medicare Linked Analysis

**DOI:** 10.7759/cureus.567

**Published:** 2016-04-12

**Authors:** Mark Amsbaugh, Beatrice Ugiliweneza, Eric Burton, Stephen Skirboll, Shiao Woo, Max Boakye

**Affiliations:** 1 Radiation Oncology, University of Louisville; 2 Department of Neurosurgery, University of Louisville; 3 Neuroncology, University of Louisville; 4 Department of Neurosurgery, Stanford University School of Medicine

**Keywords:** meningioma, radiosurgery, adjuvant radiotherapy

## Abstract

Background: The role of adjuvant stereotactic radiosurgery (SRS) and fractionated radiotherapy (XRT) are unknown in patients with resected meningiomas.

Objective: To identify patterns of care and outcomes of adjuvant radiotherapy for meningiomas in the Linked Surveillance, Epidemiology, and End Results (SEER) Medicare data.

Methods: A total of 1,964 patients older than 66 years included in the SEER-Medicare data, who were diagnosed with meningioma, and underwent craniotomy were included for analysis.

Results: Patients were less likely to receive adjuvant therapy if they were older than 75 (OR 0.730, 95% CI 0.548-0.973), female sex (OR 0.731, 95% CI 0.547-0.978), or unmarried (OR 0.692, 95% CI 0.515-0.929). Patients were more likely to receive adjuvant treatment for Grade II/III tumors (OR 5.586, 95% CI 2.135-13.589), tumors over 5 cm (OR 1.850, 95% CI 1.332-2.567), or partial resection (OR 3.230, 95% CI 2.327-4.484). Yearly between 2000 and 2009, 10.65 – 19.77% of patients received adjuvant therapy. Although no survival benefit was seen with the addition of adjuvant therapy (p = 0.1236), the subgroup of patients receiving SRS had a decreased risk of death compared to those receiving surgery alone (aHR 0.544, 95% CI 0.318 – 0.929).

Conclusion: Utilization of adjuvant XRT and SRS remained stable between 2000 and 2010. Male sex, young age, marriage, partial resection, Grade II/III tumors, and large tumors predicted the use of adjuvant therapy. For all patients, SRS decreased the risk of death compared to craniotomy alone.

## Introduction

Meningiomas are the most common intracranial tumors reported in the U.S., with an average annual incidence of 7.44 per 100,000 persons [1]. Although some meningiomas are diagnosed incidentally and follow a benign course, there is a subset that behaves more aggressively. The World Health Organization (WHO) in 1993 classified these more aggressive tumors based on histology as Grade II (atypical) and Grade III (malignant or anaplastic). The original classification has been updated in 2000, and again in 2007, to provide clearer definitions for inclusion into the higher risk groups [2]. While the treatment for less aggressive tumors is often surgical resection or observation, the optimal approach for WHO Grade II and III meningiomas is less well-defined [3]. Despite the fact that meningiomas are the most common primary brain tumor, there is a of lack of prospective and randomized clinical data to guide treatment, and the role of adjuvant therapy for meningiomas remains controversial.

The extent of surgical resection correlates with overall outcomes for meningiomas and is the most important determinant in the prevention of recurrence. Incomplete resections of malignant meningiomas lead to poor outcomes when used as a solitary treatment modality [4]. Retrospective studies have demonstrated improved local control rates with postoperative external beam radiotherapy (XRT) [4-7]. Based on these studies, post-operative XRT is often included in the adjuvant setting for WHO Grade II and III meningiomas, despite the lack of prospective randomized data [8].

There is increasing interest in using single fraction stereotactic radiosurgery (SRS) for patients with high-risk meningiomas after surgical resection. Radiosurgery may offer greater patient convenience, less adverse effects, and similar local control to traditional XRT techniques [9]. While it has been used for some time in the definitive or salvage settings for inoperable patients, reports of adjuvant SRS for resected lesions are rare [9-12]. 

The Surveillance, Epidemiology, and End Results (SEER) database, run by the National Cancer Institute, collects and publishes incidence and survival data from population-based cancer registries covering approximately 28% of the US population [13]. Data such as patient demographics, tumor site, morphology, stage at diagnosis, first treatment course, and follow-up are collected. The SEER database officially added non-malignant brain tumors in 2004 as a result of the Benign Brain Tumor Cancer Registries Act, but contains some cases recorded before that year [3]. Medicare data contains claims for Medicare beneficiaries (Americans older than 65 years or with end stage renal disease or eligible disabled individuals). These data contain healthcare resources use, procedure and diagnosis information as well as demographics. The combination of these large databases creates a powerful tool for examining patient treatment and outcomes for cancer Medicare population allowing for more detail of patients’ treatments to be examined.

We examine the SEER database combined with Medicare claims data in order to characterize patterns of adjuvant therapy following surgical resection of meningiomas between 2000 and 2009. Furthermore, we seek to identify differences in survival and repeat craniotomy between no adjuvant therapy, adjuvant SRS, and adjuvant XRT.

## Materials and methods

### Patient selection

Patients aged 66 years and older with a diagnosis of meningioma (IDC-O 9530-9534, 9537-9539) of the brain (topology codes C71.1-C71.6, C71.8-C71.9) or cerebral meninges (topology codes C70.0, C70.1, C70.9) with a WHO Grade of I, II, III, or unknown were identified in the linked SEER – Medicare records from 2000 - 2009. Patients were included only if they underwent craniotomy (ICD-9: 01.20-01.25, 01.31, 01.32, 01.31, 01.32, 01.39, 01.51, 01.53, 01.59; CPT-4: 61512, 61519) within three months after diagnosis. Only cases with confirmed histology were retained. Included patients had both part A and part B Medicare and were not a member of a health maintenance organization one month from diagnosis to either death or end of the study (Dec 31, 2009). The date of diagnosis was obtained from SEER data. To correct for the SEER dates (which are provided in month and year), the date was imputed to the 15^th^ of the month and adjustments were made to account for the 15-day error. Patients were followed up from diagnosis to either death or end of the study.

Exclusion criteria included: history of another cancer diagnosis in the data years 1997-2009, history of brain metastases any time in the follow-up period, and history of prior treatment with both XRT and SRS. Patients were grouped according to whether they received XRT (CPT-4 77427), SRS (CPT-4 77431, 77432, 77432), or neither following craniotomy. Treatment was considered adjuvant if received within six months of surgery.

### Statistical analyses

Continuous independent variables were age and Gagne Comorbidity Score (a comorbidity measure that combines the Charlson index and the Elixhauser measure) [14]. Categorical independent variables were: gender, race (white, black, other), marital status (married, unmarried/unknown), laterality (right, left, other), WHO tumor Grade (I, II/III, unknown), extent of resection (local excision, partial excision, gross total excision), diagnosis year, and tumor size (0-49 cm, 50+ cm, unknown). The outcome variables were: treatment group, survival time, and time to secondary surgery.

Continuous variables were summarized with means and standard deviation and univariately compared with the Mann-Whitney U test. Categorical variables were summarized with count and percentages and univariately compared with the Chi-square test. Time to event was analyzed with Kaplan-Meier methods and log-rank test. Multivariate comparisons, which included all the independent variables, were conducted with logistic regression for the treatment choice and proportional hazard models for time-to-event outcomes. All statistical analyses were performed using SAS version 9.3 (SAS Institute, Cary, North Carolina).

## Results

A total of 1,964 patients were included for analysis. Patient characteristics according to treatment groups are shown in Table 1. The majority of patients (n = 1701) received no adjuvant radiotherapy following meningioma resection. For patients receiving adjuvant radiotherapy, XRT was a more common treatment strategy (n = 175) than SRS (n = 88).

**Table 1:** Patient characteristics by treatment group RT*: Patients in the adjuvant therapy group had either traditional fractioned radiotherapy (XRT, n: 175) or stereotactic radiosurgery (SRS, n: 88).

		Patient Characteristics				Predictors of
		Total	RT*	No RT	p-value	RT* over no RT
Variable		(n=1964)	(n=263)	(n=1701)		Odds Ratio (95%CI)
Age, n (%)					0.0300	
	66-74	1058 (53.87)	158 (60.08)	900 (52.91)		Reference
	75+	906 (46.13)	105 (39.92)	801 (47.09)		0.730 (0.548-0.973)
Gender, n (%)					0.0006	
	Male	633 (32.23)	109 (41.44)	524 (30.81)		Reference
	Female	1331 (67.77)	154 (58.56)	1177 (69.19)		0.731 (0.547-0.978)
Race, n (%)					0.7113	
	White	1615 (82.23)	214 (81.37)	1401 (82.36)		Reference
	Black	166 (8.45)	21 (7.98)	145 (8.52)		0.865 (0.515-1.453)
	Other	183 (9.32)	28 (10.65)	155 (9.11)		1.006 (0.636-1.589)
Marital status, n (%)					0.0013	
	Married	1044 (53.16)	164 (62.36)	880 (51.73)		Reference
	Unmarried/unknown	920 (46.84)	99 (37.64)	821 (48.27)		0.692 (0.515-0.929)
Laterality, n (%)					0.4254	
	Right	764 (38.90)	96 (36.50)	668 (39.27)		Reference
	Left	773 (39.36)	102 (38.78)	671 (39.45)		1.073 (0.786-1.465)
	Other/Unknown	427 (21.74)	65 (24.71)	362 (21.28)		0.846 (0.573-1.248)
Grade					<.0001	
	Grade I	136 (6.92)	11 (4.18)	125 (7.35)		Reference
	Grade II/III	46 (2.34)	18 (6.84)	28 (1.65)		5.586 (2.135-13.589)
	Unknown	1782 (90.73)	234 (88.97)	1548 (91.01)		1.836 (0.944-3.589)
Surgery extent, n (%)					<.0001	
	Local excision	524 (26.68)	59 (22.43)	465 (27.34)		Reference
	Partial excision	352 (17.92)	92 (34.98)	260 (15.29)		1.312 (0.912-1.889)
	Gross total excision	1088(55.40)	112 (42.59)	976 (57.38)		3.230 (2.327-4.484)
Diagnosis year					<.0001	
	2000-2003	57 (2.90)	28 (10.65)	29 (1.70)		Reference
	2004	279 (14.21)	33 (12.55)	246 (14.46)		0.131 (0.064-0.268)
	2005	326 (16.60)	52 (19.77)	274 (16.11)		0.214 (0.108-0.424)
	2006	328 (16.70)	38 (14.45)	290 (17.05)		0.154 (0.076-0.312)
	2007	327 (16.65)	38 (15.45)	289 (16.99)		0.139 (0.069-0.282)
	2008	316 (16.09)	43 (16.35)	273 (16.05)		0.172 (0.085-0.346)
	2009	331 (16.85)	31 (11.79)	300 (17.64)		0.102 (0.049-0.211)
Tumor size, n (%)					<.0001	
	0-49 cm	928 (47.25)	93 (35.36)	835 (49.09)		Reference
	50+ cm	520 (26.48)	96 (36.50)	424 (24.93)		1.850 (1.332-2.567)
	unknown	516 (26.27)	74 (28.14)	442 (25.98)		1.316 (0.931-1.860)
Gagne comorbidity score						
	Mean (SD)	0.56 (1.23)	0.62 (1.41)	0.55 (1.20)		One unit increase
	Median (Q1-Q3)	0.31 (-0.20, 1.00)	0.33 (-0.20, 1.16)	0.30 (-0.20, 1.00)	0.5080	1.005 (0.902-1.120)

### Patterns of care

**Figure 1:**
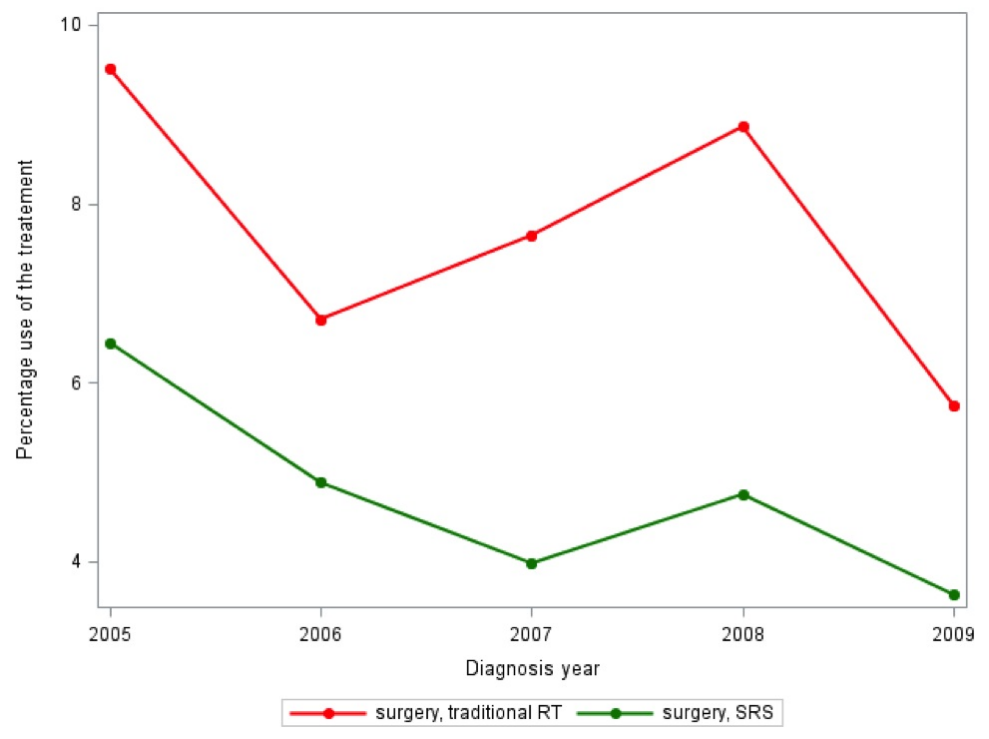
Trend graph for the use of SRS and XRT from 2005-2009 in SEER-Medicare.

**Figure 2:**
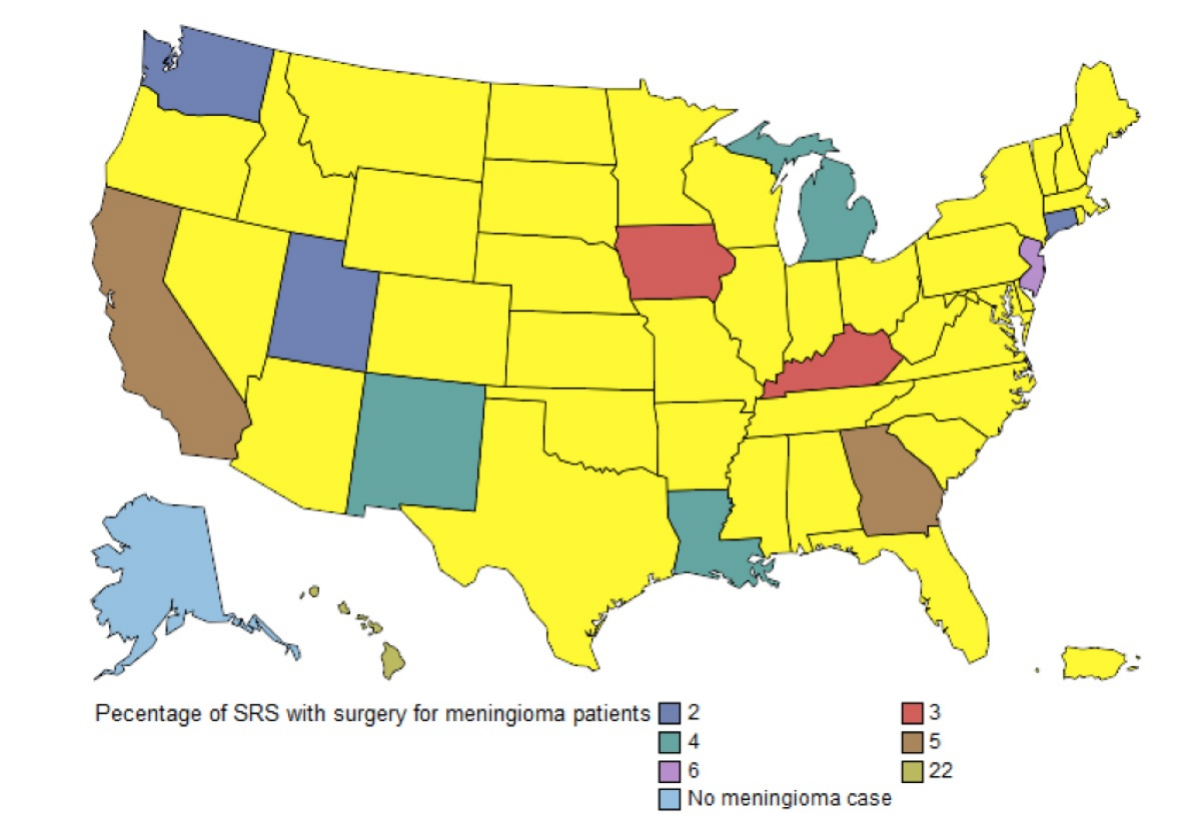
Rates of SRS use for meningioma in SEER-Medicare 2000-2010. States in yellow are not part of SEER.

Utilization of adjuvant therapy following surgical resection of meningioma remained stable over the study period. Between 2000 and 2009, 10.65 – 19.77% of patients received adjuvant therapy. Of patients receiving adjuvant therapy, SRS utilization was the lowest between 2000-2004 (18.03%). It increased to 28.32% in 2005, and since that time has remained relatively stable (31.79% – 33.46%). Figure 1 shows trends of adjuvant treatment in the meningioma population from 2000 – 2009. Figure 2 shows SRS utilization by SEER state.

Patients were less likely to receive adjuvant therapy if they were older than 75 (OR 0.730, 95% CI 0.548-0.973), female sex (OR 0.731, 95% CI 0.547-0.978), or if they were unmarried or the marital status was unknown (OR 0.692, 95% CI 0.515-0.929). Patients were more likely to receive adjuvant treatment for Grade II/III tumors (OR 5.586, 95% CI 2.135-13.589), tumors over 5 cm (OR 1.850, 95% CI 1.332-2.567), or partial resection (OR 3.230, 95% CI 2.327-4.484). No difference was seen in race (p = 0.711), location of the tumor (p = 0.425), or Gagne Comorbidity Score (p = 0.508). Treatment by degree of surgical resection is shown in Table 2.

**Table 2:** Treatment by surgery resection

	Surgery Extent		
Treatment	Local excision (N = 524)	Partial excision (N=352)	Gross or total excision (N=1088)
Traditional RT n (%)	37 (7.06)	61 (17.33)	77 (7.08)
SRS n (%)	22 (4.20)	31 (8.81)	35 (3.22)
Either of the above n (%)	59 (11.26)	92 (26.14)	112 (10.29)
No RT n (%)	465 (88.74)	260 (73.86)	976 (89.71)

### Clinical outcomes

**Figure 3:**
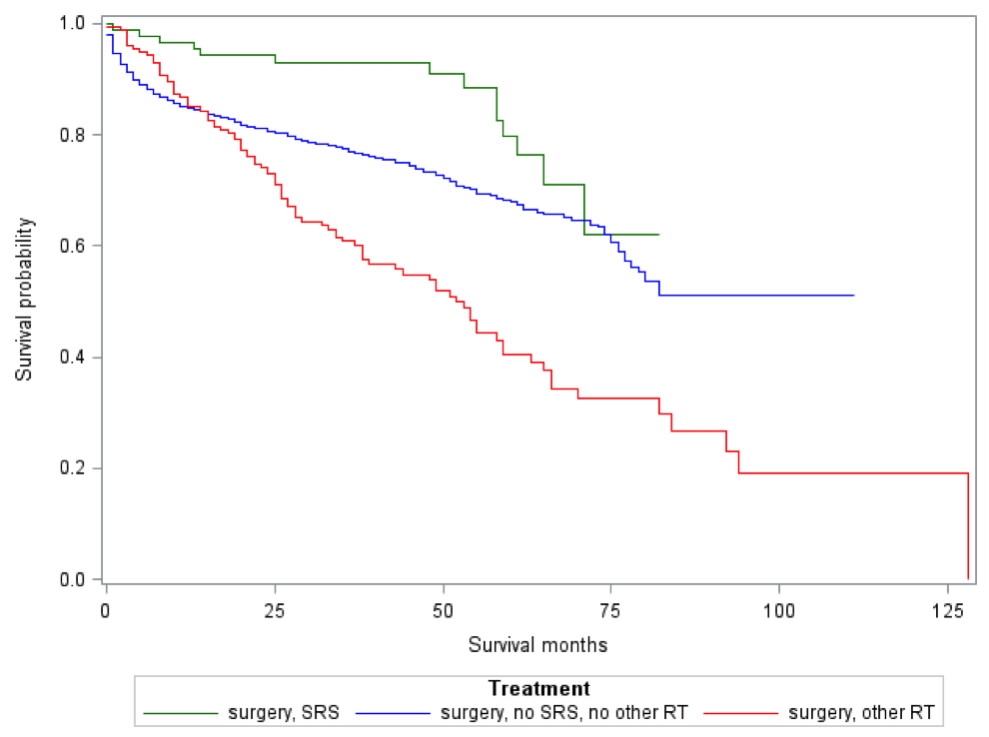
Survival analysis by adjuvant treatment type

Patients with more adverse prognostic factors were more commonly treated with adjuvant therapy forming two distinct groups. Age, female sex, race, marital status, laterality, tumor grade, tumor size, year of diagnosis, extent of surgery, and Gagne Comorbidity Score affected survival. When controlling for these factors, adjuvant therapy had no effect on survival (aHR 1.158, 95% CI 0.917 – 1.463). Kaplan-Meir survival estimates are shown in Figure 3. On univariate analysis (not considering severity of disease), patients receiving traditional radiotherapy following surgery had worse survival than those treated with SRS or no additional treatment (p <0.001). On multivariate analysis, adjuvant SRS reduced the risk of death in comparison to no adjuvant radiotherapy (aHR 0.544 95% CI 0.318 – 0.929). 

Patients with a gross total resection had a median survival of 94 months. Patients with a less than gross total resection without SRS had a median survival of 77 months compared to 71 months for those who received adjuvant SRS. Despite this difference, when controlling for adverse prognostic factors with multivariate analysis, patients who underwent less than a gross total resection and did not receive SRS had an increased risk of death compared to those who received SRS (aHR: 1.934 with 95% CI: 0.992-3.771).

Repeat craniotomy was used as surrogate marker for local tumor control. Patients who underwent adjuvant SRS had a lower rate or reoperation compared to either adjuvant RT or no adjuvant therapy (27.27 vs. 50.86 vs. 37.27%, p <0.001). There was no difference in time to second surgery between patients receiving adjuvant therapy (SRS or traditional RT) and no additional therapy (p = 0.396).

## Discussion

Our findings indicate the use of adjuvant therapy remained stable from 2000-2009. Less than 20% of patients included in the SEER data received adjuvant radiotherapy with either XRT or SRS. Of patients receiving adjuvant therapy, XRT was a more common adjuvant therapy than SRS. Stessin and colleagues examined the SEER database for all cases of non-benign meningiomas (WHO Grade II or III) and found that 37% of patients received adjuvant external beam radiotherapy [8]. Patients receiving SRS were specifically excluded from their analysis, as were patients with unknown or WHO Grade I meningiomas accounting for the difference with our data. The fraction of patients receiving adjuvant radiotherapy, even with Grade II or III meningiomas, remains low likely because strong randomized evidence to guide care decisions does not exist. For example, a recent study from two institutions looking at 158 patients with Grade II (atypical) meningiomas reported that only 15% of these patients received post-operative radiotherapy, either XRT or SRS, despite 31% of the patients had undergone a subtotal resection [15].

As expected, patients with higher grade tumors, larger tumors, or incomplete resections were more likely to receive adjuvant therapy. However, our data suggests patients who were older, women, or unmarried (or marital status unknown) were less likely to receive adjuvant therapy than their peers. This was independent of medical comorbidities or tumor characteristics. It may be that XRT is withheld more for older patients due to at least the potential for neurocognitive side effects of XRT in patients with meningiomas. Stessin and colleagues had previously noted that older patients were less likely to receive adjuvant XRT, but did not identify differences in marital status or sex, likely because of their smaller sample size. Taken together, these findings suggest potential access to care issues.

For WHO Grade I meningiomas, subtotal resection has previously been associated with inferior recurrence/progression-free survival [16]. Several retrospective series have demonstrated that adjuvant radiotherapy may overcome this effect [2, 17-22]. Adjuvant radiotherapy is commonly offered to patients after gross total and subtotal resection of WHO Grade II/III meningiomas because of decreasing progression-free survival with increasing tumor grade.

Approximately 27% of patients receiving adjuvant SRS required repeat craniotomy, lower than in all other groups. Since SEER does not contain data on local control, repeat craniotomy was used as a surrogate marker for local progression. This approach has some advantages such as the ability to ascertain the possible effect of adjuvant therapy on local control, but craniotomy after adjuvant therapy may be done for other reasons including non-oncologic indications. Patients who received adjuvant XRT had a rate of repeat craniotomy over 50%, almost twice that of SRS. Our study therefore suggests SRS may provide a local control benefit over XRT following resection of meningiomas. However, because this study examines population level data with a surrogate endpoint, more individual patient level studies will be needed to confirm the effectiveness of SRS.

Although limited data is available examining SRS in the adjuvant setting, the available literature may suggest a dose response for adjuvant XRT. No benefit was seen in a report by Goyal and colleagues, in which patients were treated with an adjuvant dose of 54 Gy [23]. However when higher doses were used in reports by Aghi and colleagues [5] and Park and colleagues [24], an improvement in local control was seen. It is unclear if adjuvant SRS improves local control by taking advantage of this possible dose response or through another mechanism. Our data did not allow examination of dose response effects.

The question of adjuvant radiotherapy’s effect on survival is complicated. While it is clear that it increases progression-free survival in high grade tumors, the literature is mixed on overall survival [4-5, 10, 25]. As has been previously described, we demonstrated decreased survival in patients who received adjuvant XRT. Stessin and colleagues examined adjuvant XRT in patients with resected, non-benign meningiomas [8]. 657 patients included in the SEER database between 2000 and 2008 were included for analysis; 244 had received adjuvant XRT. When patients with Grade II, III, and unknown were included in the analysis, patients treated with XRT had increased risk of death from any cause (HR 1.392 p = 0.034) [8]. This was thought to be a result of variables, which remained unaccounted for. Stessin and colleagues, reanalyzed data removing patients without recorded tumor grade and found no correlation between XRT and survival (p = 0.184) [8]. In our study, we sought to correct for some of the unknown cofounders likely causing this unexpected finding by using linked Medicare claims data. Our findings regarding adjuvant XRT were consistent with Stessin, as this survival detriment persists despite controlling for patient medical comorbidities. However, when patient data was re-analyzed only including tumors of known grade, no statistically significant difference was observed.

There is limited data regarding adjuvant SRS in the literature [6, 9, 26]. While adjuvant SRS has been shown to improve progression-free survival in WHO Grade II/III meningiomas, we believe that our data is the first to show a decreased risk of death in patients receiving adjuvant SRS compared to those treated with surgery alone [26]. Furthermore, in patients with incomplete resection, this benefit of SRS was seen compared to either XRT or no additional treatment.

Our study has several limitations. The use of the SEER-Medicare data allows for outcomes and patterns of care to be analyzed for a large number of patients; however, SEER represents only a fraction of patients treated for a given tumor in the United States due to constraints of data collection and funding. Despite this, SEER is a well established database for examining patterns of care and patient outcomes. It should also be noted that the SEER data represents only a first treatment course, data about subsequent recurrence is not available. Even within the data available for first treatment course, radiation dose, fraction, and treatment delivery are not available. Important prognostic factors, such as tumor grade, are variably recorded. Over 80% of patients in our study did not have a recorded tumor grade. This is consistent with other published research examining adjuvant radiotherapy outcomes in the SEER database for patients treated with meningioma [8]. Despite only 20% of the patients having a recorded tumor grade, the relative percentages of patient with Grade I (75%) and Grade II/III (25%) meningiomas in our study are similar to the percentages reported in other studies [27]. Overall survival is well represented, but data on local failure or progression of disease is more difficult to parse out. Surrogate measures (such as repeat craniotomy in this study) must be used. Another limitation is accuracy of coded data, which is an issue in all retrospective database cases. The use of combined SEER and Medicare billing data can correct for some of these covariables (such as medical comorbidities); it cannot account for others such as a large percentage of patients with an unknown grade. Our study is an analysis of registry data, and we are therefore unable to perform a randomized comparison of SRS and XRT, making our data potentially subject to selection bias issues. Although the observed survival benefit of SRS over external beam radiotherapy persisted on multivariate analysis, it may be a result of selection bias, the higher availability of SRS at centers of excellence during this time, or other unknown covariates.

## Conclusions

Usage of adjuvant radiotherapy including XRT and SRS has remained stable between 2000 and 2009 in the SEER database. Our study highlights that women and patients who are unmarried or of unknown marital status are less likely to receive adjuvant therapy for resected meningiomas. In the SEER database, patients receiving SRS had better survival and fewer repeat craniotomies than those receiving surgery alone regardless of the extent of resection. In patients treated with a partial resection, patients receiving SRS had better survival than patients receiving adjuvant XRT. This suggests SRS may be the treatment of choice for adjuvant treatment of meningiomas. Care must be taken when interpreting these results because of the limitations of the SEER-Medicare database. Future prospective clinical trials are needed to better define the role of adjuvant XRT or SRS in patients with resected meningiomas.
